# Psychiatric emergencies during, after, and before the COVID-19 lockdown: what happened to our patients? A naturalistic observational study

**DOI:** 10.1186/s12991-022-00408-z

**Published:** 2022-07-30

**Authors:** Martina Brandizzi, Annalivia Polselli, Valentina Corigliano, Stefano Maria Tamorri, Paola Venturini, Antonella Azzoni, Silvia Grasso, Antonio Onofri, Salvatore Pesce, Fiammetta Romani, Gian Marco Polselli, Alberto Forte

**Affiliations:** 1 Department of Psychiatry, ASL Roma 1, Lungotevere in Sassia, 1, 00193 Rome, Italy; 2 Department of Economics, University of Essex, Colchester, UK; 3 Psychiatry Residency Training Program, Faculty of Medicine and Psychology, Sapienza University of Rome, Rome, Italy; 4 School of Psychotherapy, Psicoterapia Training School (PTS), Rome, Italy

**Keywords:** COVID 19, Lockdown, Severe mental illness, Psychiatric emergency, Psychiatric consultation, Emergency department

## Abstract

**Background:**

Despite concerns on mental health problems related to lockdowns, recent reports revealed a reduction in psychiatric admissions in Emergency Departments (ED) during the lockdown period compared with the previous year in several countries. Most of the existing studies focused on the first lockdown not considering the different phases of the COVID-19 crisis. The present study aimed to analyze differences in ED admission for psychiatric consultation during three different phases of the COVID-19 health crisis in Italy.

**Methods:**

Information on ED admission for psychiatric consultations were retrospectively collected at the ED of the Santo Spirito Hospital in Rome (Italy), and compared between the three periods: the lockdown (March–June 2020) and the post-lockdown period (June 2020–June 2021) compared to the pre-lockdown (January 2019–March 2020). Multinomial logistic regression was used to assess the risk of accessing ED for psychiatric consultation before, during, after the lockdown.

**Results:**

Three thousand and eight hundred seventy-one ED psychiatric consultations were collected. A significant reduction of psychiatric consultations in ED during the lockdown period and the post-lockdown (H 762,45; *p* < 0.001) was documented. Multinomial logistic regression analysis showed that compared to pre-lockdown during the lockdown and post-lockdown patients were more likely to be men (RRR 1.52; 95% CI 1.10–2.12) and more often diagnosed with non-severe mental illnesses (nSMI) (relative risk ratio [RRR] 1.53, 95% CI 1.10–2.15; and 1.72, 95% CI 1.42–2.08); during the lockdown, patients were also more often diagnosed with alcohol/substance abuse (A&S) (RRR 1.70; 95% CI 1.10–2.65).

**Conclusions:**

Several changes in the clinical characteristics of psychiatric consultations during and after the lockdown emerged from the present study; nSMI and A&S abuse patients were more likely to present at the ED in the lockdown and post-lockdown periods while SMI patients appeared to be less likely. These may inform clinicians and future preventive strategies among community mental health services.

## Background

Increased levels of psychological distress and onset of mental disorders are commonly reported after mass disasters and pandemics, including post-traumatic stress, major depression, and suicide, [1, 2]. Recently published cross-sectional studies and meta-analyses reported significant psychological effects of the COVID-19 outbreak with a rising prevalence of depression, anxiety, sleep problems, PTSD on COVID-19 patients, healthcare workers [3–7] as well as among the general population [8], affecting particularly children and adolescents [9, 10]. On the other hand, a recent meta-analysis of longitudinal studies highlighted that the effects of the first national lockdown on mental health in the general population were small and heterogeneous, suggesting that positive psychological functioning, well-being, life satisfaction, and perception of social support were not affected by restrictions [11].

In line with these results and despite rising concerns of increased mental health problems during the pandemic, several studies highlighted a reduction of help-seeking behavior for psychiatric problems [12]; also, psychiatric hospitalizations appeared to be reduced during the lockdown period [13]. Recent reports on the impact of the COVID-19 pandemic on psychiatric admissions to Emergency Departments (ED) revealed a reduction in psychiatric admissions during the lockdown period compared with the previous year in several countries [14–19]. Even if the reduction in ED accesses might be associated with periods of lockdown or confinement it was observed also in countries where the restrictions imposed were markedly less strict [20]. Most of the existing studies focused on the first lockdown and presented only cross-sectional data with one time-point of observation, without comparison within the different phases of the COVID-19 crisis. In Italy, one of the first and more heavily affected European countries by the COVID-19 pandemic, a reduction of ED accesses for psychiatric consultation was found, ranging from 50 [21] to 37% in the first phase of the lockdown period (March 8–May 4th/18th 2020), and a reduction of 11,2% during the second phase of the lockdown (18th May–June 30th, 2020) [22] compared with the same periods in 2019. A study considering a longer period of observation (March 1st–August 31st, 2020) found a persisting reduction in monthly ED access for psychiatric consultation compared with 2019 [23]. Similar results emerged from a Swedish study reporting data from March 2020 to December 2020 and comparing them with the same period in 2019 and 2018 [20]. Also, the majority of studies reported a reduction of more severe mental illnesses [16] and an increase in less severe mental disorders and alcohol and substance abuse [18].

To the best of our knowledge, no studies focused on ED access during the late phase of the pandemic; analyzing the ED visits during this phase might inform on the effects of prolonged exposure to isolation, fear of contagion, government restrictions, and secondary consequences (social, economic, relational) of the pandemic among a clinical population. In particular, evidence is lacking on changes in the clinical characteristics of psychiatric consultations during and after the lockdown. Previous studies on past epidemics showed an increase in post-traumatic stress disorder, major depression, and self-injurious behavior only several months after the end of the emergency phase [24].

Given the lack of studies comparing the different phases of the COVID-19 health crisis concerning the ED consultations, the present study aimed to analyze differences in mental health emergency admissions to EDs during different phases of the lockdown comparing them with those of a pre-lockdown period. According to previous reports, we hypothesized a reduction in ED psychiatric consultations during the lockdown phase, mostly constituted by less severe psychopathological conditions and alcohol and substance abuse-related disorders. We also hypothesized that the number of psychiatric consultations would increase in the post-lockdown phase because of the effects of prolonged restrictions and secondary effects of the pandemic.

## Material and methods

We conducted a retrospective analysis on data collected from the Emergency Department (ED) of the “Santo Spirito Hospital” in Rome covering a large metropolitan area of Rome served by the *Local Health Agency ASL Roma 1*. The hospital attends a catchment area of 350,000 inhabitants.

Information on ED psychiatric consultations of patients was retrospectively collected between January 1st, 2019 and June 30th, 2021. Access to this database was provided to us through the Hospital DataWarehouse, which guarantees that the content of the information transmitted is anonymous and complies with European legal and ethical provisions. We requested data extraction from this database for all patients who were admitted from the following periods; between 8th March and 14th June 2020, which was considered the *lockdown period* of the COVID-19 pandemic coinciding with the major lockdown phases (1 and 2), between June 15th 2020 and June 30th 2021 which was identified as the *post-lockdown period* of the pandemic. These two periods were compared with a *pre-lockdown period* before the COVID-19 outbreak, from January 1st, 2019 to March 7th, 2019. The study received institutional approval and data were retrieved from the clinical administrative databases of the Local Health Agency and analyzed using an anonymous patient identifier following Data Protection Act (*EU Regulation 679/2016*). All patients provided informed consent. Data on the number of ED access, psychiatric consultations, and re-admissions were collected. Socio-demographics information (age, sex, nationality, local health agency of residency), and clinical information (mode and reasons for accessing, triage classification, diagnosis, length of stay in ED) of admissions for psychiatric consultations were analyzed.

### Measures

Diagnoses were assigned on a clinical basis and coded according to ICD 9-CM criteria. Following previous studies [16], psychiatric diagnoses were grouped into three major groups as severe mental illnesses (ICD-IXCM codes: 295, 296, 297, 298, 299, henceforth SMIs), non-severe mental illnesses (ICD-IXCM codes: 290, 293, 294, 300–302, 306–319, henceforth nSMI), and alcohol and substance use disorder (ICD-IXCM codes:291, 292, 303, 304, 305, henceforth A&S). We also identified frequent attendees of the ED, defined as individuals presenting three or more times within the study period (January 2019–June 2021) [25].

### Statistical analysis

Categorical and continuous variables were analyzed and compared between the three study periods (*lockdown period, post-lockdown period, pre-lockdown period*). Table 1 provides information on the statistical difference of each categorical (socio-demographic and clinic) variable over the three periods using a Pearson Chi-squared test for the hypothesis of independence of frequencies among the periods. For continuous variables, the statistical difference of population ranks between the three periods is obtained with the nonparametric Kruskal–Wallis *H* test. The Shapiro–Francia test was conducted to check whether variables are normally distributed and showed that the tested continuous variables are not normally distributed.

**Table 1:** Comparison of number of admissions for psychiatric consultation in ED and socio-demographic and clinical characteristics of subjects accessing during lockdown, pre-lockdown and post-lockdown

Variables	Total	Pre-lockdown	Lockdown	Post-lockdown	Pearson Chi2	Kruskal–Wallis	Shapiro–Francia
Admission in ED							
Total number of admission for any health problem in ED	67.358	43.201	3.713	20.444			
Total number of patients	2739	1423	274	1042			
Total number of psych cons in ED	3873	2033	368	1472			
Total number of admissions in psychiatric ward	924	566	93	265			
% of psych cons on ED presentations M (SD)		4.87 (0.96)	9.13 (0.67)	7.24 (0.71)		2884.46***	11.15***
ED admission per month M (SD)		3053.56 (315.12)	1352.37 (282.64)	1691.68 (299.98)		2788.88***	13.68***
ED admission with psych cons per month M (SD)		147.08 (34.22)	103.14 (34.41)	118.89 (24.56)		762.45***	8.97***
Admissions in psychiatric ward per month M (SD)		40.46 (7.44)	25.7 (6.56)	25.96 (11.82)		334.25***	7.73***
Re-admissions in ED for psych cons per month M (SD)		61.74 (17.93)	44.96 (16.07)	50.17 (10.62)		476.39***	10.28***
Socio-demographic variables							
Age M (SD)		44.49 (16.2)	43.72 (15.85)	44.31 (16.72)		0.54	9.85***
Age group *N* (%)					12.02*		
Below 35	1224	618 (30.4)	115 (31.17)	491 (33.38)			
36–49	1157	630 (30.99)	118 (31.98)	409 (27.8)			
Over 50	1364	712 (35.02)	119 (32.25)	533 (36.23)			
Unknown	128	73 (3.59)	17 (4.61)	38 (2.58)			
Gender *N* (%)					7.76***		
Female	1670	906 (44.56)	136 (36.86)	628 (42.66)			
Male	2204	1127 (55.44)	233 (63.14)	844 (57.34)			
Country of origin *N* (%)					1.98		
Italy	2918	1515 (74.52)	276 (74.8)	1127 (76.56)			
Overseas	956	518 (25.48)	93 (25.2)	345 (23.44)			
Clinic variables							
LOS M(SD)		6.26 (9.37)	8.29 (10.05)	11.92 (21.55)		266.12***	17.63***
Type of disease *N* (%)					48.08***		
SMI	1301	767 (37.73)	110 (29.81)	424 (29.16)			
nSMI	1218	573 (28.18)	126 (34.15)	519 (35.69)			
A&S	346	161 (7.92)	49 (13.28)	136 (9.35)			
Frequent attendee *N* (%)					1.69		
Yes	507	268 (14.07)	47 (12.74)	192 (13.04)			
No	3359	1747 (85.93)	332 (87.26)	1280 (86.96)			
Local Health Agency *N* (%)					14.83***		
Local Health Agency Rome 1	1860	1023 (50.32)	218 (59.08)	816 (55.43)			
Other	1817	1010 (49.68)	151 (40.92)	656 (44.57)			
Mode of arrival *N* (%)					28.83***		
Ambulance	2611	1304 (64.14)	285 (77.24)	1022 (69.43)			
Other	1263	729 (35.86)	84 (22.76)	450 (30.57)			
Triage classification *N* (%)					26.78***		
Major/urgent emergency	2144	1205 (59.27)	186 (50.41)	753 (51.15)			
Minor/non-emergency	1730	828 (40.73)	183 (49.59)	719 (48.85)			
Reason for accessing *N* (%)					17.26***		
Pyschomotor agitation	2470	1236 (60.8)	239 (64.77)	995 (67.6)			
Other	1404	797 (39.2)	130 (35.23)	477 (32.40)			
Psychiatric diagnosis *N* (%)					67.36***		
Schizophrenia and other psychosis	1020	605 (29.76)	78 (21.14)	337 (23.18)			
Mania and bipolar disorders	103	52 (2.56)	11 (2.98)	40 (2.75)			
Depression	209	120 (5.9)	24 (6.5)	65 (4.47)			
Neurosis and somatoform disorders	665	328 (16.13)	56 (15.18)	281 (19.33)			
Personality and behavioral disorders	96	46 (2.26)	15 (4.07)	35 (2.41)			
Alcholism and substance use disorders	346	161 (7.92)	49 (13.28)	136 (9.35)			
Organic mental disorders	90	35 (1.72)	5 (1.36)	50 (3.44)			
Other mental disorders	336	154 (7.58)	47 (12.74)	135 (9.28)			
No mental disorders	991	532 (26.17)	84 (22.76)	375 (25.79)			

Shapiro–Francia test tests whether a continuous variable is normally distributed; Kruskal–Wallis test is for statistical equality with continuous variables that are not normally distributed; Frequent attendee: subject with 3 or more admission in ED for Psych consultation within the period in analysis

*ED* Emergency Department, *Psych cons* Psychiatric Consultation, *LOS* length of stay in ED (hours from admission to discharge), *SMI* severe mental illness, *nSMI* not severe mental illness, *A&S* alcohol and substance abuse disorder

^***^p < 0.01, **p < 0.05, *p < 0.1. Pearson test is for statistical equality with categorical variables

Multinomial logistic regression was used to assess the risk (or likelihood) of accessing ED for psychiatric consultation before, during, after the lockdown, taking as reference period the pre-lockdown. The variables included in the regression analysis are those significant to the bivariate analysis. Age, nationality, and gender were included to adjust for socio-demographic characteristics. We also performed a forward stepwise approach for the selection of the most predictive model, adding socio-demographic and clinic controls one at a time to the most basic model with just the main predictor and the constant term (see Table 2). The most predictive models (i.e., Models 5 and 7) were chosen based on both the lowest information criteria (AIC and BIC) and the highest pseudo-R^2^. The statistical analysis was performed using the statistical software STATA/MP 17 for PC. The level of significance was set at *p* ≤ 0.05 (two-tailed).

**Table 2:** Forward stepwise analysis for the model selection

	AIC	BIC	Pseudo-R2	Set of variables included in model
Model (1)	5330	5366	0.01	Constant term and type of disorder (SMI, nSMI, A&S)
Model (2)	5331	5426	0.01	Model (1) with socio-demographic variables
Model (3)	4898	5004	0.05	Model (2) with number of hours from admission to discharge
Model (4)	4866	4996	0.05	Model (3) with controls for triage and mode of arrival
Model (5)	4795	4949	0.07	Model (4) with controls for reason for accessing and local health agency
Model (6)	4789	4955	0.07	Model (5) with binary control for frequent attendees
Model (7)	4786	4975	0.07	Model (5) with interaction term between type of disease and frequent attendees

Socio-demographic variables include age groups, country of origin, and the binary variable for gender (male)

*SMI* severe mental illness, *nSMI* not severe mental illness, *A&S* alcohol and substance abuse disorder

## Results

### Psychiatric consultations and ED accesses

We collected data on 3,873 ED psychiatric consultations relative to 2,739 patients admitted to ED between January 2019 and June 2021. Over the three study periods, 2,033 psychiatric consultations were registered in the pre-lockdown period, 368 during the lockdown period, and 1,472 during the post-lockdown period (see Table 1). The average number of psychiatric consultations per month was significantly lower during the lockdown period and the post-lockdown compared with pre-lockdown (*H* 762.45; *p* < 0.01) as well as the mean number of monthly ED readmission for psychiatric consultations (*H* 476.39, *p* < 0.01). Compared to pre-lockdown, the average monthly number of referrals to ED for any health reason was also significantly lower during the pandemic decreasing by around 56% during the lockdown and by approximately 45% during the post-lockdown (*H* 2788.88, *p* < 0.001), but the percentage of psychiatric consultations over total ED accesses was higher during the lockdown and post-lockdown (*H* 2884.46; *p* < 0.01).

### Socio-demographic variables

There were no significant differences in age and nationality between the three study periods. When age is grouped into four categories (below 35, 36–49, over 50 and patients with unknown age—who were not identified as being unable or incapable to provide an ID—)(see Table 1), the percentage of patients aged  < 35 and 50 + increased in post-lockdown compared to pre-lockdown whereas the percentages of patients aged 35–49 and those whose age is unknown decreased in post-lockdown compared to pre-lockdown. These differences were not significant (Χ^2^ 12.02; *p* < 0.1). Regarding gender, differences between men and women across the three periods are statistically significant (*Χ*^2^ 7.76; *p* < 0.01). Overall, male patients tend to present to ED more frequently than women, and their rates increased during the lockdown compared to pre-lockdown (from 55.44 to 63.14%).

### Clinical variables

Regarding clinical characteristics, there was a significant increase in the average length of stay (LOS) from admission to discharge during the lockdown and particularly in the post-lockdown was almost double (*H* 266.12; *p* < 0.01) compared to pre-lockdown. We also observed an increase in the ambulance use unlike other ways of accessing (e.g., autonomous, private transport, police, etc.) in the lockdown (*χ*^2^ 28.83; *p* < 0.01) compared to pre-lockdown, and a significant reduction of more urgent triage categories with a corresponding increase in non- and semi-urgent presentations (*χ*^2^ 26.78; *p* < 0.01) during the lockdown and post-lockdown period. During the lockdown and the post-lockdown, the majority of patients accessing ED for psychiatric consultation were belonging to the Local Health Agency Rome 1 area compared with the pre-lockdown (*χ*^2^ 14.83; *p* < 0.01).

Psychomotor agitation was the main reason for psychiatric consultation over the three study periods, but it rose by about 4% during the lockdown and 7% during the post-lockdown (*χ*^2^ 17.26; *p* < 0.01).

We also found that the most common psychiatric diagnoses in ED over the three study periods were (see Table 1): schizophrenia and other non-organic psychosis, Neurotic and somatoform disorders, and Alcohol and Substance abuse. *Schizophrenia* and *other non-organic psychosis* declined from 29.76% in pre-lockdown to 21,14% during the lockdown and to 23,18% in the post-lockdown. Conversely, diagnoses for *Alcohol and Substance abuse* grew during the lockdown and in the post-lockdown (respectively, 13.3% vs 9,35%) compared to pre-lockdown (7.9%) Diagnosis for *Neurotic and somatoform disorders* became more common during the post-lockdown compared to pre-lockdown (from 16.1 to 19.3%; with *χ*^2^: 67.36; *p* < 0.001). This trend persisted when diagnoses were grouped into major categories (i.e., SMIs, non-SMIs, A&S, and other non-psychiatric disorders) with a significant group difference (*χ*^2^ 48.08; *p* < 0.001). Compared to the pre-lockdown period we observed that SMIs were about 8% less frequent during lockdown and post-lockdown; nSMIs were around 6% and 7% more frequent in lockdown and post-lockdown; A&S abuse disorders increased by 6% during the lockdown and by 3% during the post-lockdown.

### Multinomial logistic regression

Table 3 displays the results of the main statistical model estimated using a multinomial logistic regression methodology. Clinic and socio-demographic variables are included as controls in the multinomial logistic regression, where the outcome of interest is the probability of accessing the ED for a psychiatric consultation in one of the three periods (i.e., lockdown, pre-lockdown, post-lockdown). Compared to the pre-lockdown, during the lockdown and post-lockdown patients were more often diagnosed with nSMI (relative risk ratio [RRR] 1.53, 95% CI 1.10–2.15; and 1.72, 95% CI 1.42–2.08); during the lockdown, patients were also more often diagnosed with A&S (RRR 1.70; 95% CI 1.10–2.65). In addition, compared with pre-lockdown during the lockdown and post-lockdown period patients were more likely to access for psychomotor agitation (respectively, RRR 1.41, 95% CI 1.03–1.93 and RRR 2.26, 95% CI 1.81–2.83), to arrive by ambulance (respectively, RRR 2.21, 95% CI 1.53–3.18, and RRR 1.36, 95% CI 1.09–1.69), to be registered to Local Health Agency Rome 1 (RRR 1.58; 95% CI 1.11–2.25; RRR 1.31, 95% CI 1.05–1.64), and stay longer in the emergency room (RRR 1.05, 95% CI 1.02–1.07; RRR 1.08, 95% CI 1.06–1.10). Also, they were less likely to access for major urgencies or emergencies (RRR 0.55, 95% CI 0.40–0.76; RRR 0.63, 95% CI 0.52–0.77). Furthermore, during the lockdown, they were more likely to be men (RRR 1.52; 95% CI 1.10–2.12). Compared to pre-lockdown during the post-lockdown patients aged 36–49 were less likely to be admitted for a psychiatric consultation compared to younger patients aged below 35 years (RRR 0.76; 95% CI 0.58–0.99). No statistical significance in nationality was found between the three study periods.

**Table 3:** Multinomial logistic regression analysis of the relationships between potential explanatory variables and ED admissions for psychiatric consultation during the lockdown and post-lockdown periods

Clinical variables	Lockdown			Post-lockdown		
Reference group: pre-lockdown	RRR	(95% CI)	*p*-values	RRR	(95% CI)	*p*-values
Type of disease (base cat.: SMI)						
nSMI	1.53**	(1.10–2.15)	0.013	1.72***	(1.42–2.08)	0.000
A&S	1.70**	(1.10–2.65)	0.018	1.30	(0.92–1.85)	0.135
Triage (base cat.: major/urgent emergency)	0.55***	(0.40–0.76)	0.000	0.63***	(0.52–0.77)	0.000
Reason for accessing (base cat.: psychomotor agitation)	1.41**	(1.03–1.93)	0.030	2.26***	(1.81–2.83)	0.000
Mode of arrival (base cat.: ambulance)	2.21***	(1.53–3.18)	0.000	1.36***	(1.09–1.69)	0.006
Local Health Agency (base cat.: Local Health Agency Rome 1)	1.58**	(1.11–2.25)	0.010	1.31**	(1.05–1.64)	0.018
LOS	1.05***	(1.02–1.07)	0.000	1.08***	(1.06–1.10)	0.000
Socio-demographic variables						
Age (base cat.: below 35 years)						
36–49	0.97	(0.63–1.49)	0.877	0.76**	(0.58–0.99)	0.046
over 50	0.82	(0.54–1.23)	0.332	0.79*	(0.60–1.02)	0.072
unknown	1.55	(0.72–3.35)	0.261	0.66	(0.37–1.19)	0.165
Nationality ((base cat.: Italian)	1.07	(0.72–1.61)	0.726	1.13	(0.86–1.48)	0.370
Gender ((base cat.: Male)	1.52**	(1.10–2.12)	0.012	1.11	(0.89–1.38)	0.346

*LOS* length of stay in ED (hours from admission to discharge), *SMI* severe mental illness, *nSMI* not severe mental illness, *A&S* alcohol and substance abuse disorder

^***^p < 0.01, **p < 0.05, *p < 0.1

We further examined the moderating role of frequent attendees in the association between the type of disease (SMI, nSMI, A&S) and the access to ED for a psychiatric consultation over the three study periods (Table 4). We found that during the post-lockdown frequent attendees were more likely to be diagnosed with SMI and nSMI disorders and not with A&S abuse compared with the pre-lockdown period (RRR 1.59, 95% CI 0.95–2.67 and RRR 1.93; 95% CI 1.04–3.59).

**Table 4:** Multinomial logistic regression analysis of the relationships between potential explanatory variables and ED admissions for psychiatric consultation during the lockdown and post-lockdown periods with the interaction between frequent attendees and the type of disease

Reference group: pre-lockdown	Lockdown			Post-lockdown		
Clinical variables	RRR	(95% CI)	*p*-values	RRR	(95% CI)	*p*-values
Type of disease (base cat.: SMI)						
nSMI	1.42**	(1.01–1.98)	0.041	1.71***	(1.39–2.09)	0.000
A&S	1.52*	(0.95–2.44)	0.079	1.41**	(1.01–1.99)	0.047
Type of disease x frequent attendee						
SMI x frequent attendee	0.46	(0.18–1.21)	0.117	1.59*	(0.95–2.67)	0.077
nSMI x frequent attendee	1.37	(0.55–3.42)	0.496	1.93**	(1.04–3.59)	0.037
A&S x frequent attendee	1.43	(0.52–3.94)	0.489	0.68	(0.15–3.11)	0.615
Triage (base cat.: major/urgent emergency)	0.55***	(0.40–0.76)	0.000	0.64***	(0.53–0.78)	0.000
Reason for accessing (base cat.: psychomotor agitation)	1.40**	(1.02–1.91)	0.037	2.25***	(1.80–2.81)	0.000
Mode of arrival (base cat.: ambulance)	2.17***	(1.51–3.13)	0.000	1.40***	(1.12–1.75)	0.003
Local Health Agency (base cat.: Local Health Agency Rome 1)	1.58**	(1.11–2.24)	0.012	1.32**	(1.05–1.66)	0.016
LOS	1.05***	(1.02–1.07)	0.000	1.08***	(1.06–1.10)	0.000
Socio-demographic variables						
Age (base cat.: below 35 years						
36–49	0.96	(0.63–1.47)	0.860	0.76**	(0.58–1.00)	0.049
Over 50	0.82	(0.54–1.24)	0.353	0.77*	(0.58–1.02)	0.065
Unknown	1.59	(0.74–3.42)	0.235	0.68	(0.38–1.22)	0.199
Nationality (Italian)	1.06	(0.71–1.59)	0.763	1.11	(0.85–1.46)	0.431
Gender (male)	1.53***	(1.11–2.11)	0.010	1.07	(0.86–1.34)	0.541
Constant omitted						
Robust CI in parenthesis						

Frequent attendee: subject with 3 or more admission in ED for psychiatric consultation within the period in analysis

*LOS* length of stay in ED (hours from admission to discharge), *SMI* severe mental illness, *nSMI* not severe mental illness, *A&S* alcohol and substance abuse disorder

^***^p < 0.01, **p < 0.05, *p < 0.1

## Discussion

To the best of our knowledge, this is the first study evaluating ED access for psychiatric consultations in Italy before, during, and after the lockdown. The present study aimed to analyze the longitudinal variations on psychiatric emergencies in a hospital located at the center of Rome from January 2019 to June 2021, considering different phases of the lockdown as well as the period after the lockdown, and the changes in clinical features of subjects accessing ED for a mental health problem. We found a significant reduction in monthly ED psychiatric consultations in the lockdown persisting during the post-lockdown period compared with the pre-lockdown period, as well as different clinical presentations of patients between the three study periods. Namely, we found during the lockdown and post-lockdown compared to the pre-lockdown a remarkable drop (8%) of psychiatric consultation associated with SMIs that paralleled an increase of A&S abuse disorders particularly in the lockdown and of nSMI during the post-lockdown.

Previous studies focused on the impact of the first lockdown on EDs psychiatric consultations with data from a limited time frame [22, 23, 26, 27], and no study has yet investigated the late phase of the pandemic and the long-term effects of prolonged exposure to the pandemic on psychiatric emergencies. Even if most of the present findings might be related to the government restrictions to movement and to the fear of contagion, our results are consistent with evidence from other countries, suggesting a plausible general effect of the COVID-19 crisis on severe mental illnesses; while a severe mental health crisis was expected [28, 29], a general reduction in help-seeking behaviors [28–31] and psychiatric emergencies were found [14–16, 27]. In line with previous Italian studies [22, 23], although the described reduction in EDs psychiatric consultations was higher in the lockdown period, there was a persisting reduction in the post-lockdown period as well.

Confirming data from a recent German report [32], we found a reduction in ED access for any type of health problem in the lockdown period, persisting in the post-lockdown. Interestingly, the percentage of psychiatric consultations over total ED accesses was higher during the lockdown and the post-lockdown period. These findings, in line with those emerging from another study considering data from Italian ED (Lombardy) [22], demonstrate that the need for mental health problems remained high despite the containment measures compared to other emergencies. Focusing on demographic characteristics, nationality did not contribute to the risk of access to ED for a psychiatric consultation. We did find evidence of age differences in the probability of accessing the ED for psychiatric consultations only in post-lockdown with respect to pre-lockdown. In particular, adults (aged 36–49 and 50 + , respectively) had a minor risk to request a psychiatric consultation than young adults (aged below 35). This is in line with other studies that have highlighted an increased demand in consultations for adolescents, and several studies highlighted that adolescents and young adults were most affected by restrictions, particularly those with previous mental health difficulties [10].

Moreover, in line with previous studies, we found an increased likelihood to access ED for nSMI during lockdown and post-lockdown. Frequent attendees in the post-lockdown tended to be diagnosed more often with nSMI disorders as well as with SMI. The drastic reduction in levels of care that was found during the lockdown period might have affected nSMI who were not prioritized by community mental health services [33].

The reduction of ED access for subjects with SMI during the lockdown and post-lockdown confirms evidence of other studies and could be explained by the fact that patients affected by psychotic disorders were already suffering from social isolation before the lockdown, thus movement restrictions and social distancing might have initially less affected them [16]. It is interesting to note that higher population density was found to be associated with fewer social contacts in patients with psychosis [34]; thus we might argue that social isolation in psychosis is not related to the availability of social contact, rather than to psychopathological characteristics of SMI. It might be argued that in the most severe psychopathological conditions such as schizophrenia, it might be more distressing the return to “normal” life and deal with social relationships than the lockdown [35].

Furthermore, we may speculate that the reduction observed in SMI could be related to a longer duration of untreated psychosis (DUP) and in the next future, more severe cases will be detected. Future studies might be aimed to address the relation between DUP and the COVID-19 crises.

Our findings also expand previous literature on ED psychiatric consultations, as increased demand for drug and substance abuse and nSMI during the pandemic was found [21], suggesting that social isolation and movement restrictions might have affected more those clinical populations rather than those affected by severe mood disorders and psychosis. This confirmed previous studies on the clinical population revealing that the COVID-19 health crisis affected mainly some clinical subgroups of patients and most severely children and adolescents [36–38]. Also, the present findings might inform future preventive strategies at the community level, and highlight changes in the nature of psychiatric epidemiology in Italy and elsewhere. This might as well inform policy makers and stakeholders, for the need of reorganization of psychiatric services in the light of the rising of substance use disorders and nSMIs as emerging acute psychopathological conditions in ED and inpatients psychiatric settings.

The increase in A&S abuse might also reflect the increases in addiction-related habits in the general population during the early phase of COVID-19 containment, paired with reduced well-being and increased stress factors [39, 40]. Also, our findings are in line with previous studies showing that abuse of drugs or alcohol increased substantially during the pandemic in several countries, and many people increased their consumption of substances to cope with isolation [41]. In addition, social distancing, isolation, and confinement may have affected people with pre-existing substance use disorders exacerbating loneliness, psychological distress, and driving them to frequent relapses [42]. Organizational difficulties in addiction services due to the pandemic could also have increased ED access for A&S abuse. Also, our findings confirmed that subjects most frequently accessing ED for psychiatric consultation during the lockdown were males with A&S-related disorders. In addition, we found that men have a greater risk to access the ED for a psychiatric consultation during the lockdown, in contrast with previous literature suggesting that women are at higher risk of suffering from lockdown measures [38, 43, 44].

Our and previous findings should not be taken as conclusive evidence that the COVID-19 pandemic does not affect subjects with SMI. Evidence on the long-term effects of prolonged or repeated lockdown is still limited. Over time, in addition to the enduring social isolation and fear of contagion, the economic and social costs of the pandemic restrictions (loss of job, closure of businesses, financial insecurity, remote work, etc.) could have affected even more mental health. To date, most studies focused on the medium-term consequences of the COVID-19 pandemic on mental health. Given the persistence of the pandemic, the question of the psychological impact of repeated or prolonged restrictions and lockdowns remains open, and more research is needed to address the long-term effects on mental health.

## Strengths and limitations

Findings from the present study might be interpreted in the light of several limitations. First, we were not able to retrieve information on pre-existing psychiatric problems when the patient was at his first admission. Further limitations might be the availability of a limited number of socio-demographic and clinical variables, as well as the lack of specific measures for the psychological distress associated with the COVID-19 period. Furthermore, with the present findings, we cannot infer the long-term effects of the lockdown and the impact of a prolonged exposition to the pandemic and its socio-economic and relational consequences on mental health remains unknown. Another limitation to consider is a lack of differences in urbanicity, as the catchment area considered in the present study takes into account only an urban area. Nonetheless, the present study relayed on data retrieved from three different phases of the pandemic, adding to the literature relevant clinical as well as epidemiological information.

## Conclusions

We evaluated ED access for psychiatric consultations in a central hospital in Rome during, after, and before the lockdowns due to the COVID-19 Health crisis. A significant reduction in monthly ED psychiatric consultations was found during the lockdown compared with the pre-lockdown period. We also found that during the lockdown and post-lockdown, patients were more likely to access ED for psychomotor agitation, less likely to access for major urgencies or emergencies, less likely to present a SMIs, and more likely to present A&S abuse. The present findings suggest several changes in the clinical characteristics of psychiatric consultations during and after the lockdown, confirming a reduction in SMIs and an increase in A&S abuse and nSMI. These findings may inform clinicians and future preventive strategies among community mental health services. Taken together, our findings suggest that patients affected by SMIs might have presented unexpected higher levels of resilience during the public health crisis and were less affected by isolation. Conversely, a higher number of substance use disorders and nSMIs were found. The effects of a prolonged exposition to the Covid-19 pandemic and its socio-economic consequences on psychiatric emergencies remain unknown, and future studies might focus on the late phase of the pandemic.

## Data Availability

Data are available upon request due to privacy reasons.
